# Bi deficiency-tuned functionality in multiferroic Bi_1-δ_Fe_0.95_Mn_0.05_O_3_ films

**DOI:** 10.1038/srep19385

**Published:** 2016-01-18

**Authors:** Jingyi Chen, Yao Wang, Hui Wang, Shuangmei Zhang, Yuan Deng

**Affiliations:** 1 School of Materials Science and Engineering, Beijing Key Laboratory for Advance Functional Materials and Thin Film Technology, Beihang University, Beijing 100191, China

## Abstract

Structural evolution and ferroelectric (FE)-to-antiferroelectric (AFE) transition behaviors were observed in Bi_1-δ_Fe_0.95_Mn_0.05_O_3_ (100)-textured films with a carefully controlled Bi deficiency concentration δ. Raman spectra revealed an orthorhombic structural transition induced by Mn substitution. The polarization-electric field hysteresis loops and capacitance-voltage loops of Bi_1-δ_Fe_0.95_Mn_0.05_O_3_ films clearly demonstrated antiferroelectric behavior with increasing δ. The responses of the domain structure of the Bi_1-δ_Fe_0.95_Mn_0.05_O_3_ film under positive and negative applied voltages directly suggested the coexistence of FE and AFE phases. The existence of (100) superstructure reflections and antiparallel displacements of the Bi atoms along the [100] direction observed by transmission electron microscopy unambiguously reveal the AFE phase. The chemical substitution-induced orthorhombic structural transition in BiFe_0.95_Mn_0.05_O_3_ film implies that as the δ concentration increases, the changes in Bi-O bonding and the stereochemical activity of Bi 6*s* lone pair affect both the ferroelectric distortion and the antiferrodistortive rotation and therefore drive the Bi_1-δ_Fe_0.95_Mn_0.05_O_3_ crystal lattice to form a PbZrO_3_-type orthorhombic phase with an AFE order. A continuing increase in Bi deficiency creates defect dipole complexes which produce an internal field leading to a preferred direction of the ferroelectric domain. The Bi deficiency in multiferroic BiFeO_3_ provides a new route by which to tune functionality.

Currently, BiFeO_3_ (BFO) is the only known single-phase multiferroic material that simultaneously shows ferroelectric (***T***_*C*_~1103 K) and antiferromagnetic (***T***_*N*_~640 K) orderings at room temperature. The fundamentally interesting physics and the flexibility of external field control using perturbations such as stress, electric and magnetic fields in the multiferroic system make BFO an attractive material^1^,^2^,^3^. Recent studies of highly strained BiFeO_3_ thin films have motivated a new round of intense research on this material due to its great application potential in lead-free probe-based data storage and actuator systems^4^,^5^. In addition to strain engineering in BFO films, chemical modification of BFO is also under active investigation to modulate the structures, reduce leakage currents, lower the coercive field or increase the magnetic ordering^6^,^7^. Rare-earth ion doping at the Bi site in BFO films was reported to lead to a structural transition from the ferroelectric rhombohedral phase to an orthorhombic phase exhibiting a double polarization hysteresis loop. Such a structural transition can be universally achieved by controlling the average ionic radius of the Bi-site cation^8^. A PbZrO_3_-like structure with antipolar order was also observed in Bi_1-x_Nd_x_FeO_3_ bulk materials^9^. Because the ferroelectricity in BFO originates from the Bi^3+^ 6s^2^ lone pair, Bi-site modification direct influences Bi-O bonding modifying the off-center displacement of the cation. Studies on Mn-doped BFO bulk materials showed that Mn substitution at the Fe site changed both structural and electronic properties, resulting in a phase transition from the *R*3*c* space group to the *Pnma* space group^10^,^11^,^12^. Recent research demonstrated that Bi was also affected by Mn substitution due to Fe 3*d*-O 2*p*-Bi 6*s* electron hybridization^11^,^12^. Thus, while modification of the Bi-site in BFO lattice tends to induce an antiferroelectric structure, structural transitions are also promoted by Mn substitution at the Fe-site.

Very recently, Kalinin and Spaldin discussed how the effects of functional ion defects in transition metal oxides may provide a new route for the control of novel functionalities^13^,^14^. It is therefore important to determine whether the same structure modification will be generated using Bi vacancies instead of heterogeneous element substitution at the Bi-site in Mn-doped BFO thin films due to the extreme ease of Bi vacancy formation in BFO. Here, we demonstrate that Bi deficiency functions as a new parameter in tuning the phase structure and ferroelectric behavior of BFO films. We carefully controlled the Bi deficiency concentration in Mn-doped (5 mol%) BFO films by utilizing the volatilization of Bi during high-temperature heat treatment. Double polarization hysteresis loops were observed in Bi-deficient Mn-doped BFO films, indicating antiferroelectric-like behavior. The origin of this antiferroelectricity-like behavior in Bi_1-δ_Fe_0.95_Mn_0.05_O_3_ film is discussed.

## Material and Methods

Mn (5 mol%) doped BFO thin films were grown on Pt/Ti/SiO_2_/Si substrates via a simple chemical solution deposition technique as reported before^15^. Because of the volatility of Bi in the annealing process, excess Bi is needed in the starting materials to compensate for the bismuth loss. Here, different contents, i.e., 2, 5, and 10 mol%, of excess Bi were chosen to adjust the Bi deficiency concentration in BFO films. The final thickness of the films is approximately 220 nm. Circular Au electrodes with a diameter of 0.2 mm were sputtered on the film surface using a shadow mask for electrical characterization.

X-ray diffraction (XRD, Rigaku D/max2500HB +/PC with Cu Kα radiation λ = 1.54056 Å in a θ/2θ mode) was used to check the crystal structures. Raman spectra were collected in backscattering geometry on RM2000 (Renishaw) with Ar^+^ laser irradiation at 514 nm focused over a 5-μm diameter area. Inductively coupled plasma mass spectrometry (ICP-MS, Thermo ICP-MS XII) was used to check the chemical compositions of the Bi_1-δ_Fe_0.95_Mn_0.05_O_3_ films. The Bi deficiency value δ is 0.034 for Bi content excess of 2 mol%, 0.028 for Bi content excess of 5 mol%, and 0.005 for Bi content excess of 10 mol% BFO films. Transmission electron microscopy (TEM, JEM-2100F, JEOL), high-resolution TEM (HRTEM) and nano-beam electron diffraction (NBED) images were taken at 200 kV. Ferroelectric hysteresis loops were tested using a standardized ferroelectric test system (Radiant Technology, Precision Workstation), and Agilent B1505A was utilized to take capacitance-voltage measurements. Domain structures and local piezoelectric coefficients were observed and measured using a piezoelectric force microscope (PFM, PISA XEI100) operating at an *ac* of 2.5 V at 17.5 kHz.

## Results and Discussion

**Figure 1:**
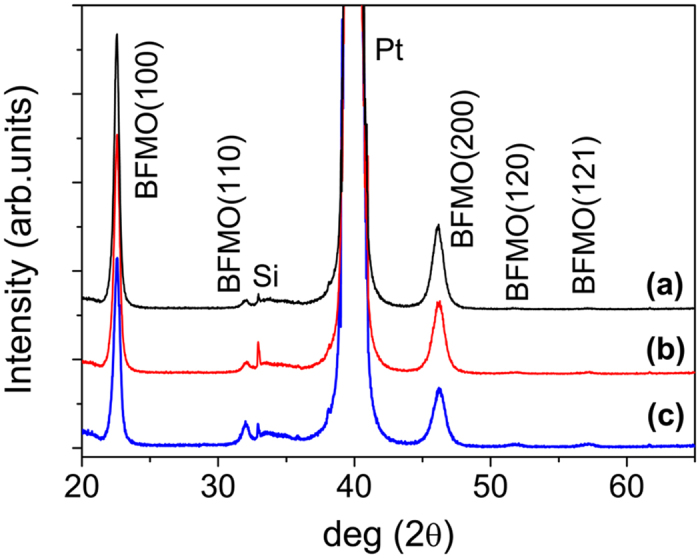
XRD patterns of Bi_1-δ_Fe_0.95_Mn_0.05_O_3_ films with different Bi deficiency contents. (**a**) δ = 0.005, (**b**) δ = 0.028, and (**c**) δ = 0.034.

Bi_1-δ_Fe_0.95_Mn_0.05_O_3_ (BFMO) films with different Bi deficiency amounts all show pure perovskite phase XRD patterns, as shown in Fig. 1. An obvious (100) plane diffraction preference is observed for all BFMO films. Although the positions of the diffraction peaks do not change, the films’ texture, the broadening of the peaks and the merging of the split peaks in the *R*3*c* structure make the XRD patterns insufficient to determine the crystal structure of these BFMO films. Therefore, further information on the details of the change in crystal symmetry of BFMO films with different Bi deficiencies is obtained by Raman spectroscopy.

**Figure 2:**
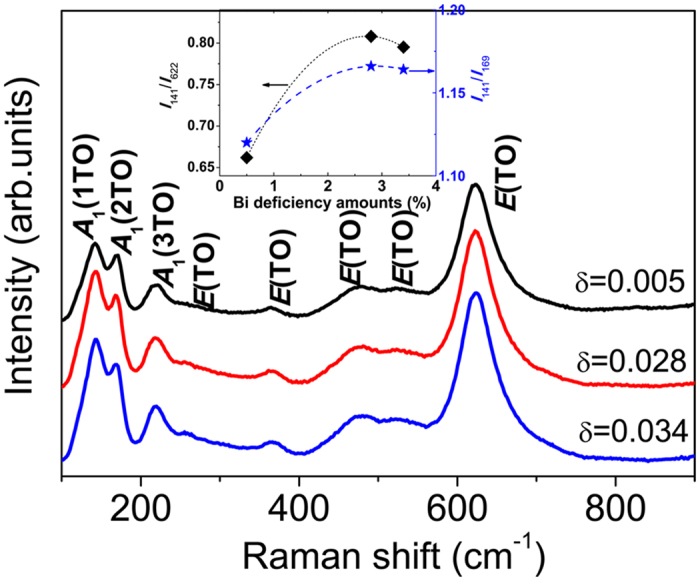
Raman spectra of Bi_1-δ_Fe_0.95_Mn_0.05_O_3_ films with different δ concentrations. The inset shows the change of intensity ratios of Raman vibrational modes at 141 cm^−1^ with 622 cm^−1^ and 169 cm^−1^, respectively, with Bi deficiency concentrations.

Eight vibration modes are clearly identified in the Raman spectra of all the BFMO films shown in Fig. 2(a). Here, the quite strong *E*(TO) vibrational modes at 622 and 480 cm^−1^ resemble those observed in orthorhombic manganite perovskites that display Jahn-Teller distortions, as noted by a recent study on BiFeO_3_-LaMnO_3_ solid solution thin films^16^. Mn ions are implicated as the main cause of octahedral distortion that induces the transition to an orthorhombic BFO structure. Thus, it is reasonable to deduce that the structural distortion arisen in BFO films due to the substitution of 5 mol% Mn shows the appearance of orthorhombic phase. Meanwhile, the characteristic peaks of rhombohedral phase still retained. Raman spectra were also used to analyze the Bi deficiency because the Raman peaks between 120 and 250 cm^−1^ are attributed to Bi-O vibrations. No Raman peak shift was observed in BFMO films with different Bi deficiency concentrations; however, the intensity ratios of the peaks changed. As shown in the inset of Fig. 2, the intensity ratio between the *A*_1_(TO) mode at 141 cm^−1^ and the *E*(TO) at 622 cm^−1^, which are the characteristic peaks of the orthorhombic phase, increases with increasing Bi deficiency; the intensity ratio between the two *A*(TO) modes at 141 cm^−1^ and 169 cm^−1^ follows the same trend. The change in Raman mode intensity ratios that represent the vibration of Bi-O bonds with the Bi deficiency amount directly confirms that Bi deficiency influences Bi-O bonding in the BFO lattice.

**Figure 3:**
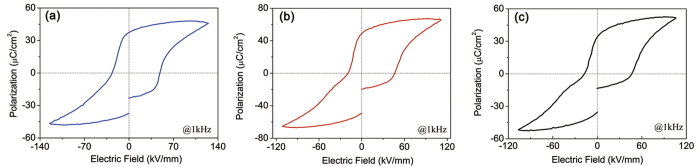
*P-E* hysteresis loops of Bi_1-δ_Fe_0.95_Mn_0.05_O_3_ films with increasing Bi deficiency contents measured at 1 kHz. (**a**) δ = 0.005, (**b**) δ = 0.028, and (**c**) δ = 0.034.

**Figure 4:**
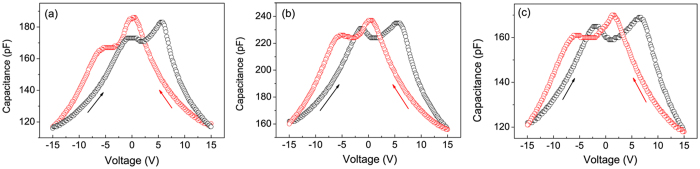
*C-V* loops of Bi_1-δ_Fe_0.95_Mn_0.05_O_3_ films with increasing Bi deficiency contents. (**a**) δ = 0.005, (**b**) δ = 0.028, and (**c**) δ = 0.034.

The electrical properties of BFMO films were examined via polarization-electrical field (*P-E*) hysteresis loops and capacitance-voltage (*C-V*) loops, as shown in Figs 3 and 4, respectively. As shown in Fig. 3(a), the BFMO film with the almost stoichiometric composition (i.e., δ = 0.005) exhibits a normal *P-E* hysteresis loop with a remanent polarization of 37 μC/cm^2^, displaying ferroelectric characteristics. The gap between the initial and the final polarizations may be due to partial domain depolarization after a presetting voltage pulse. As shown in Fig. 3(b,c), with increasing Bi deficiency concentration, i.e., δ = 0.028 and 0.034, the square-shaped P-E hysteresis loops become distorted and develop into doubled hysteresis loops that are the typical characteristic of an antiferroelectric material.

Point defects or defect dipoles have long been considered to be responsible for the observed double *P-E* loops in ferroelectrics^17^. A general microscopic mechanism was proposed by Ren to explain the strange double hysteresis in doped or irradiated ferroelectrics after aging^18^. Here, the Bi deficiency was intentionally introduced, and oxygen vacancies are inevitable in perovskite films fabricated using a chemical method. According to the defect reaction 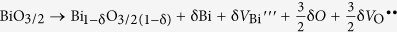, Bi deficiency 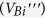 will promote the formation of an oxygen vacancy 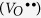. Thus, the oxygen vacancy and Bi deficiency are likely to form defect dipole complexes that will lead to domain pinning.

As seen from the *C-V* loop of a Bi-sufficient BFMO film presented in Fig. 4(a), a shoulder-peak-like plateau was observed in the process of either increasing or decreasing voltage. Similar *C*-*V* loops were reported for BFO films in a previous study and were attributed to the 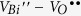
defect dipole complexes^19^. Such defect-dipole complexes produce an internal field leading to a preferred direction of the ferroelectric domain. Comparison to the BFMO film with δ = 0.005 shows that as δ increases to 0.028 and 0.034, clear double butterfly-shaped *C-V* loops showing two capacitance maxima for both increasing and decreasing voltages appear in Bi-deficient BFMO films [see Fig. 4(b,c)], again showing the typical characteristics of antiferroelectricity.

Consideration of the *C-V* loop together with the *P-E* behavior of BFMO film for a stoichiometric composition shows that defect dipole complexes alone cannot account for the all the observations. Because antiferroelectric phase transition has been proven to exist in Mn-doped BFO material, it is quite reasonable that the phase transition may be another origin of the antiferroelectric-like behavior. Belik *et al*. discovered an antiferroelectric orthorhombic PbZrO_3_-type phase in BiFe_1-x_Mn_x_O_3_ (0.15 ≤ x ≤ 0.4) solid solutions^11^. The deformation of the (Fe,Mn)O_6_ octahedra is primarily driven by the covalent Bi-O bonding and the related stereochemical activity of the lone pair. Therefore, when we increase the amount of Bi deficiency, the Bi-O bonding and the stereochemical activity of Bi 6s lone pairs in the crystal cell of the BFMO film are changed so as to drive the deformation of the (Fe,Mn)O_6_ octahedra into a structure resembling an orthorhombic PbZrO_3_-type phase.

**Figure 5:**
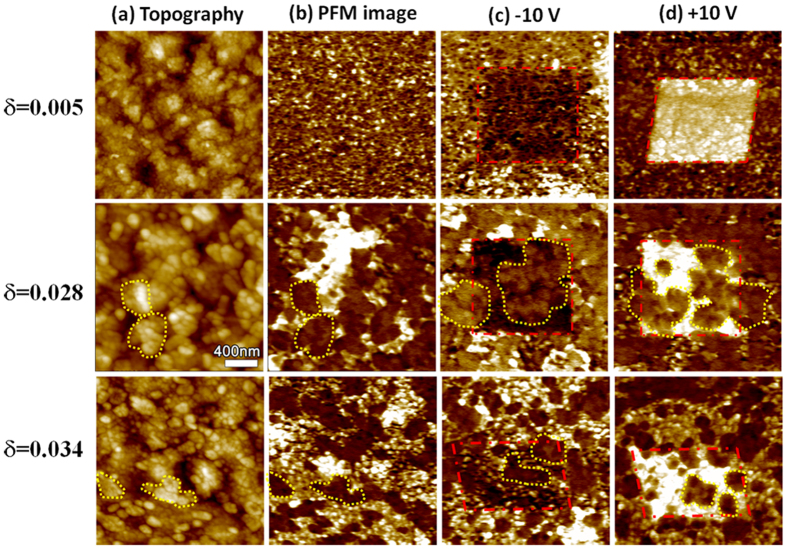
(**a**) Topography images and (**b**) OP-PFM amplitude images of Bi_1-δ_Fe_0.95_Mn_0.05_O_3_ films with the inner frame area polarized under (**c**) -10 V and (**d**) + 10 V, respectively. The size of the scanning area for all the images is 2 μm × 2 μm with the inner frame area 1 μm × 1 μm.

The dynamic responses of BFMO films to the applied electric field were investigated via the observation of domain switching behaviors. The topography and the corresponding out-of-plane (OP) PFM amplitude images of BFMO film with δ = 0.005 are presented in Fig. 5(a,b) and show that the grains of Bi-sufficient film are quite small, with the size of approximately 20 nm, and each grain contains a single domain with either an OP polarization direction (i.e., perpendicular to the film surface and pointing either downward or upward). The domain structures obtained after the film were polarized by the PFM probe in an area of 1 μm × 1 μm under negative and positive 10 V, are shown in Fig. 5(c,d), respectively, and show that all domains in the polarized area were completely switched to align to the direction of the applied electric field. However, when δ increases to 0.028 and 0.034, regions showing a near-zero piezoelectric response (circled by the dashed line) were observed in the domain structures of the BFMO films. When an external electric field is applied, the FE domains are switched to follow the external electric field orientation, while the non-responsive areas are hardly changed by the applied voltage. Moreover, we found some connection between the lack of response of the non-polarized regions and their corresponding microstructures, as shown in Fig. 5(a,b) with δ = 0.028 and 0.034, as indicated by the dashed lines. The AFE domains generally show clusters of grains with a higher topography on the film surface; this is probably caused by the lattice mismatch between the matrix FE rhombohedral phase and the transformed AFE orthorhombic phase. The PFM measurements demonstrate the formation of a complex mixture of FE and AFE phases in the Bi-deficient BFMO films, as illustrated in Fig. 5. The same phenomenon was also discussed by Cheng *et al*^20^. They found a complex nanoscale mixture bridging the Bi-rich rhombohedral and Sm-rich orthorhombic phases at the phase boundary in the Bi_1−x_Sm_x_FeO_3_ thin films. The growth of clusters of AFE regions within a FE matrix leads to a high frustration of the FE regions by the surrounding AFE matrix, resulting in an incommensuration phenomenon.

**Figure 6:**
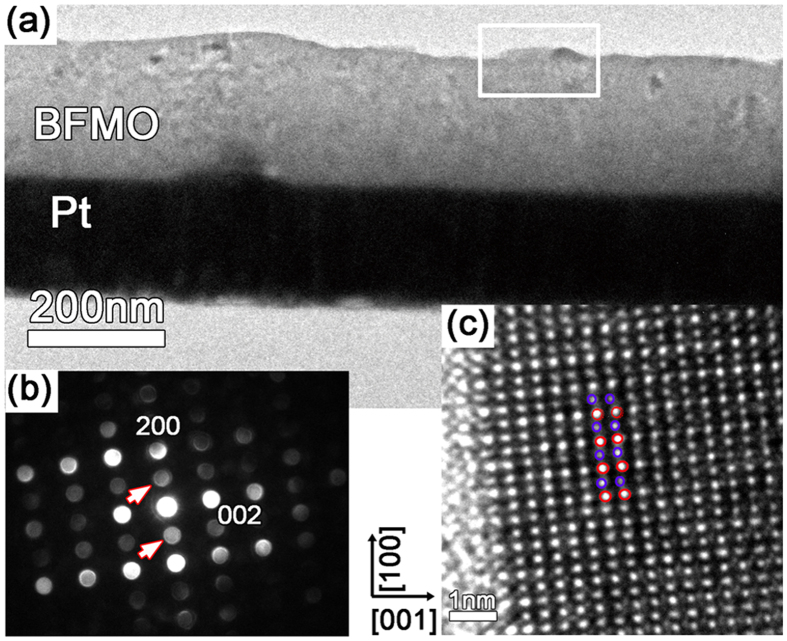
(**a**) Cross-sectional TEM image of Bi_1-δ_Fe_0.95_Mn_0.05_O_3_ film with δ = 0.028. (**b**) Nano-beam electron diffraction along <011 > zone axis of the framed region in Fig. 6(a) and the (100) superstructure reflections marked by arrows indicate the antiparallel cation displacement. (**c**) Corresponding HRTEM image of the framed region showing the alternating shifts of Bi ions left and right along the [100] direction.

To further understand the origin of the antiferroelectric-like behavior, HRTEM and electron diffraction were used to characterize the atomic arrangement of the Bi-deficient BFMO film. Figure 6(a) shows a cross-sectional TEM image of Bi_1-δ_Fe_0.95_Mn_0.05_O_3_ film with δ = 0.028. Nano-beam electron diffraction (NBED) was used to obtain the electron diffraction of the region that corresponds to the AFE domain showing a higher topography on the film surface. Figure 6(b) presents an image of NBED along the <011 > zone axis of the framed region in Fig. 6(a); the (100) superstructure reflection marked by arrows are typically directly associated with the antiphase tilting of oxygen octahedra. These reflections are attributed to the antiphase rotations of the oxygen octahedral^9^,^20^,^21^. The corresponding HRTEM image of the framed region is shown in Fig. 6(c), where the alternating shifts of Bi ions left and right along the [100] direction are indicated by blue and red circles. Antiparallel displacements of the Bi atoms along the [100] direction clearly demonstrate the presence of an antipolar order in Bi-deficient BFMO film and confirm that the film is in the AFE phase.

**Figure 7:**
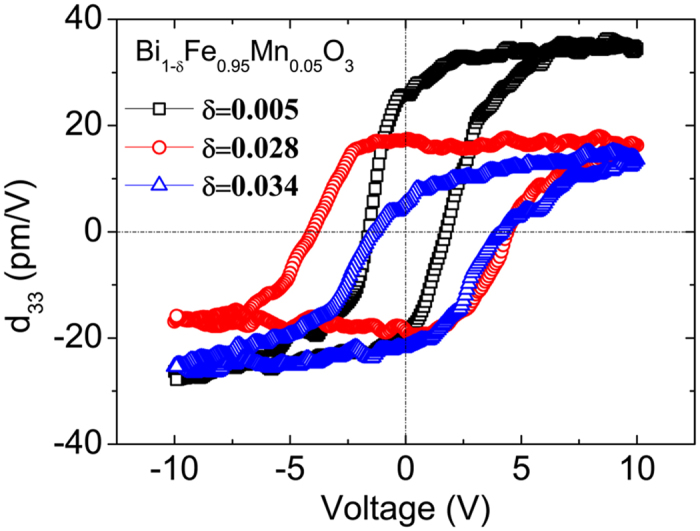
Piezoelectric coefficient *d*_33_ as a function of applied voltage for BFMO films with different Bi deficiency concentrations.

We also carried out measurements of local piezoelectric coefficients (*d*_33_) of the BFMO films, with the results shown in Fig. 7. A sharp decrease in the absolute value of *d*_33_ (from 31 pm/V for δ = 0.005 to 18 pm/V δ = 0.028 BFMO film) was observed upon the appearance of the AFE phase in the BFMO film. As Bi deficiency concentration increased to δ = 0.035, a decrease in the absolute value of *d*_33_ (~15 pm/V) A as well as an obvious shift of the hysteresis loop toward a positive bias and negative piezoelectric coefficient were observed, indicating the dominating preferred polarization direction in the film. Such observation in the *d*_33_ hysteresis loop of BFMO film with high Bi deficiency δ = 0.035 implies that the defect dipole complexes probably produce an internal field leading to a preferred direction of the ferroelectric domain. The observed domain structures for δ = 0.035 were consistent with this hypothesis, while the polarization of some domains is pre-oriented up (Fig. 7(b)) and even after the application of the maximum downward voltage (−10 V in our experiment), not all the domains are reversed downwards, as shown in Fig. 7(c); it is much easier for all the domains to orient upwards as shown in Fig. 7(d).

Therefore, the introduction of Bi deficiency tuned the structural transition such that Bi_1-δ_Fe_0.95_Mn_0.05_O_3_ films undergo a phase change from FE to AFE due to the interaction between (Fe,Mn)O_6_ octahedra and Bi-O bonding and the stereochemical activity of the Bi 6s lone pair; under high Bi deficiency concentrations, domain structures of the FE phase show a preferred polarization orientation due to the influence of Bi vacancy and oxygen vacancy defect dipole complexes.

## Conclusions

In summary, by introducing a small amount of Bi deficiency instead of heterogeneous element substitution, we obtained Bi_1-δ_Fe_0.95_Mn_0.05_O_3_ films with mixed FE and AFE phases showing double hysteresis *P-E* loops and *C-V* loops. Based on orthorhombic distortion induced by Mn substitution, Bi deficiency changes the Bi-O bonding and the stereochemical activity of Bi 6s lone pair in the crystal cell, thereby driving the formation of PbZrO_3_-type orthorhombic AFE phase. The observations of superstructure reflections and cation displacements by TEM together with the comparison between domain structures and the switching behaviors of Bi sufficient and Bi deficient Bi_1-δ_Fe_0.95_Mn_0.05_O_3_ films unambiguously identify the FE and AFE phases. Under high Bi deficiency concentration, domain structures of the FE phase are manipulated by Bi vacancy and oxygen vacancy defect dipole complexes. These results demonstrate that deficiency can be treated as a new parameter to tune the functionality of multiferroic BFO film.

## Additional Information

**How to cite this article**: Chen, J. *et al*. Bi deficiency-tuned functionality in multiferroic Bi_1-δ_Fe_0.95_Mn_0.05_O_3_ films. *Sci. Rep*. **6**, 19385; doi: 10.1038/srep19385 (2016).
